# Brain-to-brain communication during musical improvisation: a performance case study

**DOI:** 10.12688/f1000research.123515.2

**Published:** 2023-05-09

**Authors:** Mauricio A. Ramírez-Moreno, Jesús G. Cruz-Garza, Akanksha Acharya, Girija Chatufale, Woody Witt, Dan Gelok, Guillermo Reza, José L. Contreras-Vidal

**Affiliations:** 1 Mechatronics Department, Tecnologico de Monterrey, Monterrey, Nuevo Leon, 64849, Mexico; 2 Noninvasive Brain-Machine Interface Systems Laboratory, NSF IUCRC BRAIN, University of Houston, Houston, Texas, 77004, USA; 3 University of California, Los Angeles, Los Angeles, California, 90095, USA; 4 Moores School of Music, University of Houston, Houston, Texas, 77004, USA; 5 Houston Community College, Houston, Texas, 77004, USA; 6 Independent Musician, Houston, Texas, USA

**Keywords:** Brain on arts, hyperscanning, brain-to-brain synchrony, musical improvisation

## Abstract

Understanding and predicting others' actions in ecological settings is an important research goal in social neuroscience. Here, we deployed a mobile brain-body imaging (MoBI) methodology to analyze inter-brain communication between professional musicians during a live jazz performance. Specifically, bispectral analysis was conducted to assess the synchronization of scalp electroencephalographic (EEG) signals from three expert musicians during a three-part 45 minute jazz performance, during which a new musician joined every five minutes. The bispectrum was estimated for all musician dyads, electrode combinations, and five frequency bands. The results showed higher bispectrum in the beta and gamma frequency bands (13-50 Hz) when more musicians performed together, and when they played a musical phrase synchronously. Positive bispectrum amplitude changes were found approximately three seconds prior to the identified synchronized performance events suggesting preparatory cortical activity predictive of concerted behavioral action. Moreover, a higher amount of synchronized EEG activity, across electrode regions, was observed as more musicians performed, with inter-brain synchronization between the temporal, parietal, and occipital regions the most frequent. Increased synchrony between the musicians' brain activity reflects shared multi-sensory processing and movement intention in a musical improvisation task.

## Introduction

Advances in neuroengineering have fostered the development of mobile brain-body imaging (MoBI) technologies and denoising algorithms that allow the acquisition, interpretation, and decoding of brain activity from free-behaving individuals in real settings. ^1^^–^^3^ These advances have led to the development of neurofeedback systems, brain-computer interfaces (BCIs) and neuroprostheses. ^4^ These devices provide aid in the treatment of neurological disorders such as Parkinson’s disease, epilepsy and depression, ^5^^–^^7^ motor impairments, ^8^ and diminished brain functioning. ^9^ Although all of these systems are extremely helpful to patients and healthy persons, they follow an individualistic, personal-use approach. ^10^

While an understanding of an individual’s cognitive function is of utmost importance in the development of neurological treatments, the comprehension of social interactions at a neurological level is also important, as humans are social beings by nature, ^11^ and neurological disorders such as autism spectrum disorders (ASD) can affect social communication and interaction. ^12^ Furthermore, many common daily human activities are carried out in groups, e.g. at school, work, sports, creative art, and leisure. ^11^^,^^13^ Thus, research advances in social neuroscience are likely to revolutionize different fields such as entertainment, communication, education, healthcare, and social embedding, among others. ^14^ Recently, researchers have started to explore brain activity from a collective perspective, using a contemporary approach, known as hyperscanning. ^15^

### Brain synchrony assessment through hyperscanning

Hyperscanning refers to the synchronous recording of brain activity from more than one individual simultaneously, and has been implemented to study dynamic similarities or differences between the brain signals of multiple participants engaged in interactive or social tasks. ^15^ Such an approach holds promise in understanding the nature of cognitive traits during social interactions. ^15^ Recent hyperscanning studies have documented traces of shared cognition, emergent during moments of social interaction, collaboration, competition, and in educational settings. ^16^^,^^17^ The study of neural synchrony between individuals provides an insight into human connectedness and may aid in the development of treatments for social cognition disorders such as autism. ^18^ A desired outcome of hyperscanning is the development of neural biomarkers that track in real-time the quality or strength of shared cognitive states such as brain-to-brain communication, shared attention, message conveying, and high engagement during human interactions.

Indeed, recent studies on human interactions have analyzed shared brain dynamics during teamwork tasks, ^19^ and cooperative/competitive interactions. ^16^ It has been reported that neural synchronization increases when participants are interacting in cooperation, and it reduces when they are competing against each other. A hyperscanning study allowed the quantification of the synchronization between brain signals of infants and adults during gaze interactions, showing increased neural coupling during direct eye-contact. ^20^ Neural coupling between humans has also been associated to the degree of mutual pro-sociality, where higher synchronization reflects stronger social relationships, ^21^ and likeliness of interpersonal bonding. ^22^ Considering the aforementioned studies, by analyzing inter-brain activity, hyperscanning offers a quantitative assessment of the strength and quality of different types of social interactions. ^23^

Regarding neural synchrony metrics, among the most common are coherence, ^17^ phase coherence, ^16^ phase locking value (PLV) and phase locking index (PLI), ^15^ Granger causality, ^20^ correlation, ^21^ wavelet transform coherence (WTC), ^22^ graph theory, and partial directed coherence (PDC). ^23^ Bispectrum is another, more recent, metric in hyperscanning literature, ^19^^,^^24^ and offers insights on temporal, spatial and spectral levels. The bispectrum of a signal is a high order spectra that reflects the degree of temporal synchronization and phase coupling between two time series at different frequencies. ^25^ The bispectrum offers additional insight when compared to other neural synchrony metrics, as it provides a more complete intuition on phase coupling, ^19^ resonance, temporal synchronization and non-linear interactions ^26^ between any analyzed signal pair ^25^; rather than only focusing on phase-coupling or correlation. Moreover, bispectrum is feasible to implement in real-time applications, which makes it more attractive for closed loop brain-computer interface applications. ^19^

### Musical improvisation as a window to study Brain-to-brain synchrony

Studies on intra and inter neural synchrony between pairs of guitarists during musical improvisation have shown dynamical networks that connect different brain regions, depending on the situation and/or expectations, with involvement of the fronto-parietal region, as well as the somatosensory, auditory, and visual cortices. ^27^^,^^28^ The analysis of such obtained networks can be used to study the temporal dynamics of these interactions and providing a neurophysiological interpretation of the observed behavior. Considering the rich and complex interchange of cognitive processes necessary during collaborative artistic performances, its study using the hyperscanning approach is a valid approach to explore the shared neural cognitive traces that emerge from these interactions. ^29^^,^^30^

Collaborative, free musical production (improvisation) is a complex and rich form of social interaction, ^31^ it has also been described as a continuous process of generation and transformation of musical interaction, ^32^ and offers an interesting object of study for hyperscanning. Similarities between music and language have been observed previously in terms of the social interaction they entail; as described in, ^33^ a jazz improvisation can be interpreted as a conversation, and a good improvisation as a complex, meaningful conversation. Over recent years, there has been a growing interest in the study of improvisational, freely-moving, collaborative musical production in live performance settings. ^23^ Hyperscanning in the musical context can allow the observation of neural traits of dynamic processes. For example the patterns between musicians’ brain activity when performing cooperatively or not, as it has been reported that such actions create differences in their peripersonal space, ^34^ and in the rhythmical alignment of the overall performance. ^35^

This process of improvised production can be perceived as a creative act of communication: one that is complex, nuanced, and technical, integrating simultaneous cognitive processes together in real time. Musical improvisation involves complex but rapid interactions of several components, including the generation and evaluation of melodic, harmonic, and rhythmic pattern ideas on a fast time-scale within a performance. ^36^ Mobile brain-body (MoBI) imaging provides the tools for analyzing neural patterns in real-time for freely-moving participants, ^1^^,^^2^^,^^37^ with hyperscanning techniques that provide an experimental approach to assess non-verbal communication in musical performance. ^29^ During an improvised performance, musicians interact with each other, making use of different skills such as creativity, ^38^ emotional expression and perception, ^31^ self-organization, ^39^ memory retrieval and procedural memory, ^40^ and integration of visual and auditory stimuli with complex and precise motor coordination. ^41^^,^^42^ Musical improvisation can also be considered as an 'in the moment' composition, where improvisers are able to generate immediate responses to the present musical environment, to manipulate ideas and sequences in a spontaneous manner. ^43^

### Brain-to-brain synchrony between musicians in free-jazz improvisation

Among many music styles, this study is centered on jazz, which takes roots from a mixture of afrological (african-american), and eurological (classical and contemporary) music. ^44^ More specifically, the performance of “free jazz” is of interest to this study, due to its unique characteristics that convey freedom to the perfomers. It is not based on pre-defined musical components (e.g. melody, rythm and harmony), pushes boundaries of improvisational norms, avoids the existence of shared musical frameworks; and allow musicians to generate temporal coherent pieces using improvisation as its major element. ^44^

In a theoretical model to study group jazz improvisation, Biasutti and Frezza ^45^ identify the processes that are essential for creative musical improvisation: anticipation, use of repertoire, emotive communication, feedback, and flow. In Wopereis *et al.,* ^46^ 26 expert musicians provided statements about musical improvisation in two 10-min individual brainstorm sessions. The statements resulted in a 7-cluster concept map, with self-regulation as the central concept, and affect, risk-taking, ideal, basic skills, responsivity, and creation, as constituent concepts for improvisational expertise. Specifically for collaborative improvisation, monitoring, feedback, and evaluation must be performed in association with other musicians, with both generative and communicative attentional demands. ^47^ Another study on jazz improvisation remarks that shared intentions emerge on the fly, and their presence fosters acoustic and temporal coordination, as well as improving the quality of the performance, as perceived by the performers and listeners. ^13^

Recently, the predictive coding of music (PCM) model has been introduced to model how listeners form expectations which may be fulfilled or not, through perception, action, emotion and, over time, learning. ^48^ Under this model, musical interaction is guided by mutual reduction of prediction errors, evidenced by alpha-band intrabrain neural synchronization (phase-locking analysis) in a right-lateralized temporoparietal network, with a higher occurrence probability in mutually adapting dyads than in leader-leader dyads. ^49^ These models of music improvisation highlight the centrality of anticipation, self-regulation, generation and evaluation, with feedback and communication in joint performance.

### Research aims of this case study

This article aims to contribute to the understanding of brain-to-brain communication during a collaborative musical improvisation between jazz musicians. In this case, brain-to-brain communication can be understood as the existent non-verbal communication (e.g., anticipation, planning, and actions such as taking turns) between dyads of participants (jazz musicians improvising), ^50^ that can be assessed by quantifying and analyzing the synchrony between their brain signals, recorded simultaneously using the hyperscanning approach. ^51^ A jazz performance incorporates each of the five elements of musical improvisation: anticipation, feedback, use of previous repertoire, emotive communication, and coordinated flow. ^45^ Moreover, in a free jazz performance, as described in, ^52^ a continuous process of evaluation is present, where musicians can decide to maintain or change the current theme; initiate or respond to a change; and to adopt, augment, or contrast a given idea.

While improvising, musicians can elaborate over (but are not constrained to) a composition’s underlying chord structure ^53^ and theme, with variations that incorporate multiple derivations from instantaneous decisions in real-world practice. ^54^ An important aspect of jazz performance and proficiency lies in the embodied cognition and motor memory. ^55^ However, most neuroimaging studies on musical improvisation have used functional magnetic resonance imaging (fMRI). ^36^^,^^47^ Lying down in an fMRI scanner alters spatial and visual perception, ^56^ and restricts body movement, which limits the capability of fMRI studies to observe realistic musical performance given the importance of embodied cognition in the task (due to the continuous retrieval and processing of spatial, auditory, visual and somatosensory information). ^55^ Because mobile electroencephalography (EEG) does not impose movement constraints, (as compared to the ones imposed when lying down in an fMRI scan), and subsequently allows participants to naturally engage in creative production with minimal, instrumentational constraint, it may afford advantages in studying musical improvisation. ^57^^,^^58^

The current study examines the neural correlates of brain-to-brain communication of jazz musicians during collaborative musical improvisation through hyperscanning; and addresses the limited body of knowledge on collaborative musical improvisation in an ecologically-valid production, with freely-moving expert musicians, as they interact in a jazz performance with a live audience. Here, the presence of a live audience is important, as our cohort of musicians are accustomed to them, to the point that they become a relevant part of their performance. An inter-brain synchronization analysis was implemented, by estimating the bispectrum of EEG signals between musician pairs during collaborative improvisations as they performed for a live audience. Following the concept of ecological validity, the exquisite corpse method was adopted to obtain realistic collaborative improvised art pieces. ^37^^,^^57^ The exquisite corpse is a game played by surrealists, in which different artists integrate their contributions into a unique piece, taking turns to add their input in an iterative manner until completing a final piece with the contributions of all members. ^37^ Under this paradigm, the complete performance is formed by a multi-participant improvisational, free jazz piece formed by the creativity from each player.

## Methods

### Human participants

The experimental methods were approved by the Institutional Review Board of the University of Houston, and are in accordance with the Declaration of Helsinki. All participants provided written informed consent, including agreement for publication in online open-access publication of information obtained during the experiments such as data, images, audio, and video. Three male healthy adults (P _1_, P _2_ and P _3_) volunteered for this study. Musicians P _2_ and P _3_ received (formal) musical instruction for 12 and 6 years, respectively, and P _1_ (informal) for 6 years. To the date of the experiments, P _1_, P _2_ and P _3_ had 31, 38, and 26 years of experience performing music, respectively. P _2_ and P _3_ were music educators at the University of Houston at the time of the experiment. The musicians performed jazz improvisation in a public event at the Student Center of the University of Houston while wearing the MoBI technology. Musicians P _1_ and P _2_ have a jazz musical background, whereas P _3_ had a’classical music’ education. Musician P _1_ played the drums, musician P _2_ played the saxophone, and P _3_ played using a soprano saxophone. P _1_ and P _2_ had performed jazz regularly together for 6 years, P _2_ and P _3_ had performed a concert together once before, and P _1_ and P _3_ had not performed together previously. The heterogeneity between participants' experience and familiarity was not voluntary targeted; however, it led to interesting results and interpretations, presented in Results and Discussion sections.

### Equipment

High-density active electrode scalp EEG and electrooculography (EOG) recordings were obtained simultaneously for the three musicians during their musical performances. EEG data was wirelessly acquired using the 64 channel actiCAP (BP gel) electrodes along with the Brain Amp DC amplifer (actiCap system, Brain Products GmbH, Germany) at a sampling frequency of 1000 Hz. Electrode distribution follows the 10-20 international system. EEG data was online referenced to channel FCz on the superior region of the scalp. Four channels were used to record EOG data. Channels TP9 and TP10 were placed on the right and left temples, respectively, to record horizontal eye movement, whereas channels PO9 and PO10 were placed above and below the right eye, respectively, to record vertical eye movement. Electrode impedances were recorded prior to the experiment, and maintained below 60 kΩ following the manufacturer's guidelines in the Brain Vision Recorder software and User manual on impedance measurement (where impedance values above 60 kΩ are considered ‘bad’, and below 25 kΩ ‘good’). ^59^

Performances were recorded by three video cameras coupled with a Zoom H6 ( https://zoomcorp.com/) audio recorder from a frontal, superior and lateral perspective. Audio was recorded in a single stereo file at 44100 Hz. Three Sterling ST31 FET condenser microphones ( https://sterlingaudio.net/) were used to amplify the sound from each musician’s instrument during the live performance.

### Experimental design

Musicians performed three 15 minutes improvisations (trials). Each trial was divided in three 5 minutes pieces (segments). In Segment 1, one musician was performing while the other two were listening. In Segment 2, a different musician joined the first, while the remaining musician listened to the performance of the other two. In Segment 3, the third musician joined and all participants performed together until the end of the trial. In a given segment, the musicians who are performing are referred to as the “active” musicians, whereas the musicians who are not performing are the “passive” musicians. At the beginning and at the end of the experiment, three blocks comprising of an EEG impedance check, a one-minute eyes open (EO) and a one-minute eyes closed (EC) were recorded.

In each trial, the order of the musicians joining at each segment was pseudo-randomized so that each musician entered one trial as the first, second or third player. Each musician was given a visual cue to signal their start time in the piece. Between one trial and the next, there were short pauses of 3-5 minutes, in which the audience clapped and the musicians prepared for the next trial.

**Figure 1.**
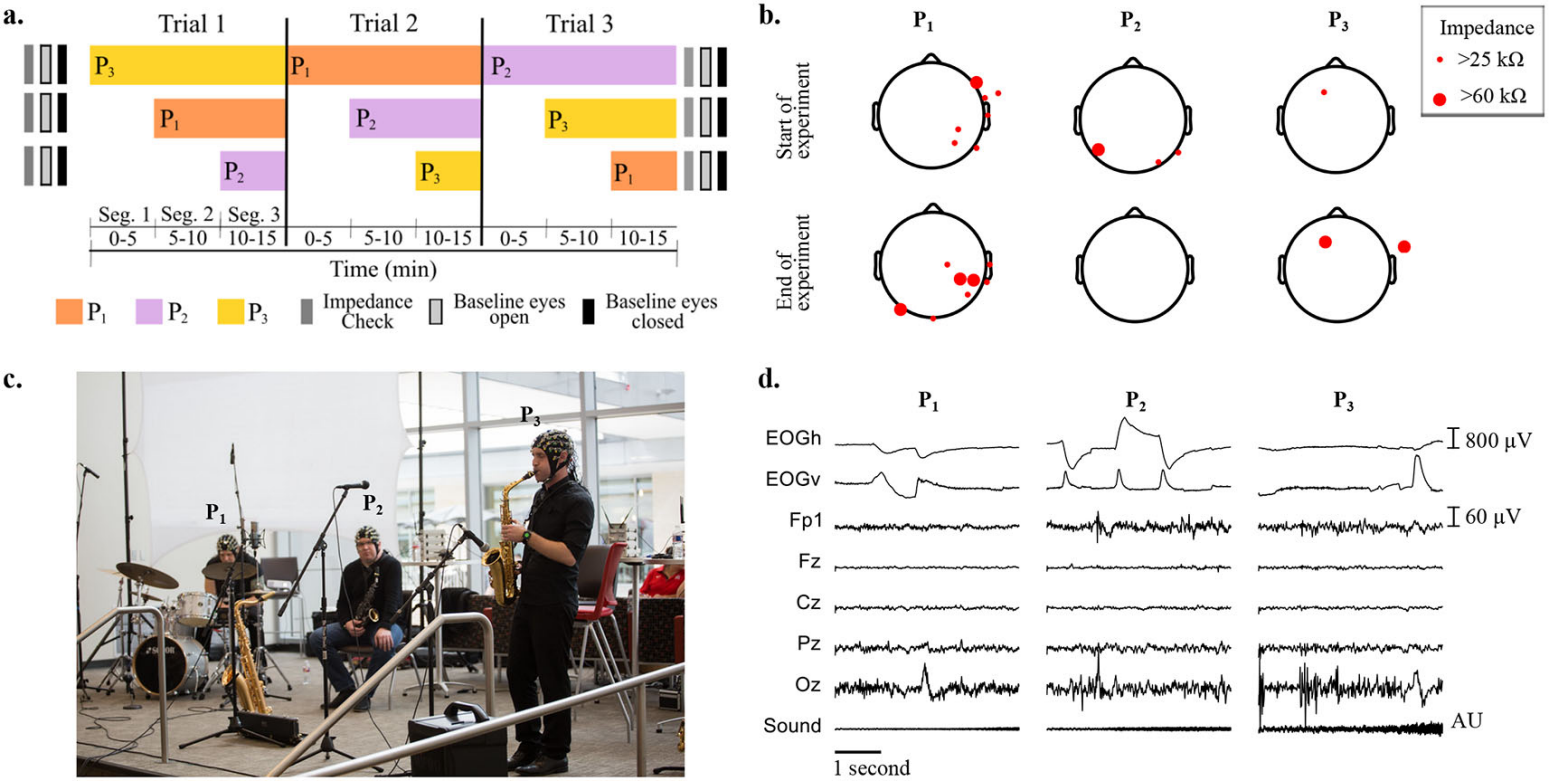
a) Impedance check, baseline (eyes open and eyes closed) measurements and performance times for each participant across the three improvisation trials. b) Impedance values larger than 25 kΩ across electroencephalographic (EEG) electrodes at the start and end of all experiments for the three participants. c) Experimental setup of musicians on stage wearing EEG caps and performing. From left to right: P _1_ (drums), P _2_ (saxophone) and P _3_ (soprano saxophone). d) Representative electrooculography (EOG), EEG and sound recordings during musical performance of the first five seconds of Trial 1.

Figure 1(a) shows the protocol for baseline measurements, and the order of musicians joining at each segment and trial. Figure 1(b) shows the locations of EEG electrodes with impedance higher than 25 kΩ at the start and at the end of the recordings, for all musicians. Figure 1(c) shows the setup of the instruments and microphones during the experiments, and the three musicians wearing the EEG caps. Figure 1(d) depicts, as an example, from top to bottom, the recorded raw EOG, EEG and audio signals obtained for the first five seconds of Segment 1 of Trial 1, when only P _3_ is performing.

Three independent raters with training in music composition annotated the data. The annotators were not familiar with the researchers nor with the musicians involved in the performances; and were tasked to write annotations of the performance independently from each other, by watching a recording of the live performance. The annotators were asked to write a short description (e.g. “players are performing in sync”, “mirroring each other”, “performing in discord”), and the time each event happened. Table 1 shows sample descriptions from the annotators during one trial of musical improvisation.

**Table 1.** Type, times and annotations of events labelled by annotators (in the audience) during Trial 1. Only synchronized performance (SP) and desynchronized performance (DP) events are presented.

Type of event	Time	Annotation
SP _1_	5:20	Drums and soprano saxophone synchronize
DP _1_	5:46	Drum solo
DP _2_	6:28	Both play unevenly
SP _2_	7:03	Mirror each other
DP _3_	7:34	Drum deviates
SP _3_	8:23	Mirror each other
DP _4_	11:07	Saxophone and soprano saxophone discord; both are trying to lead
SP _4_	11:17	Rapid, loud performing - some mirroring
DP _5_	12:01	Discord

At the beginning of each trial, video, audio and physiological signals were synchronized using manual event markers (i.e. pressing a button). Recordings from the three trials were obtained simultaneously using this procedure. Unfortunately, data transmission was interrupted from 4:25-5:25 of Trial 3 due to a loss in connection, which resulted in missing data. The events that happened in this period were therefore not included in the analyses.

### Signal preprocessing

**Figure 2.**
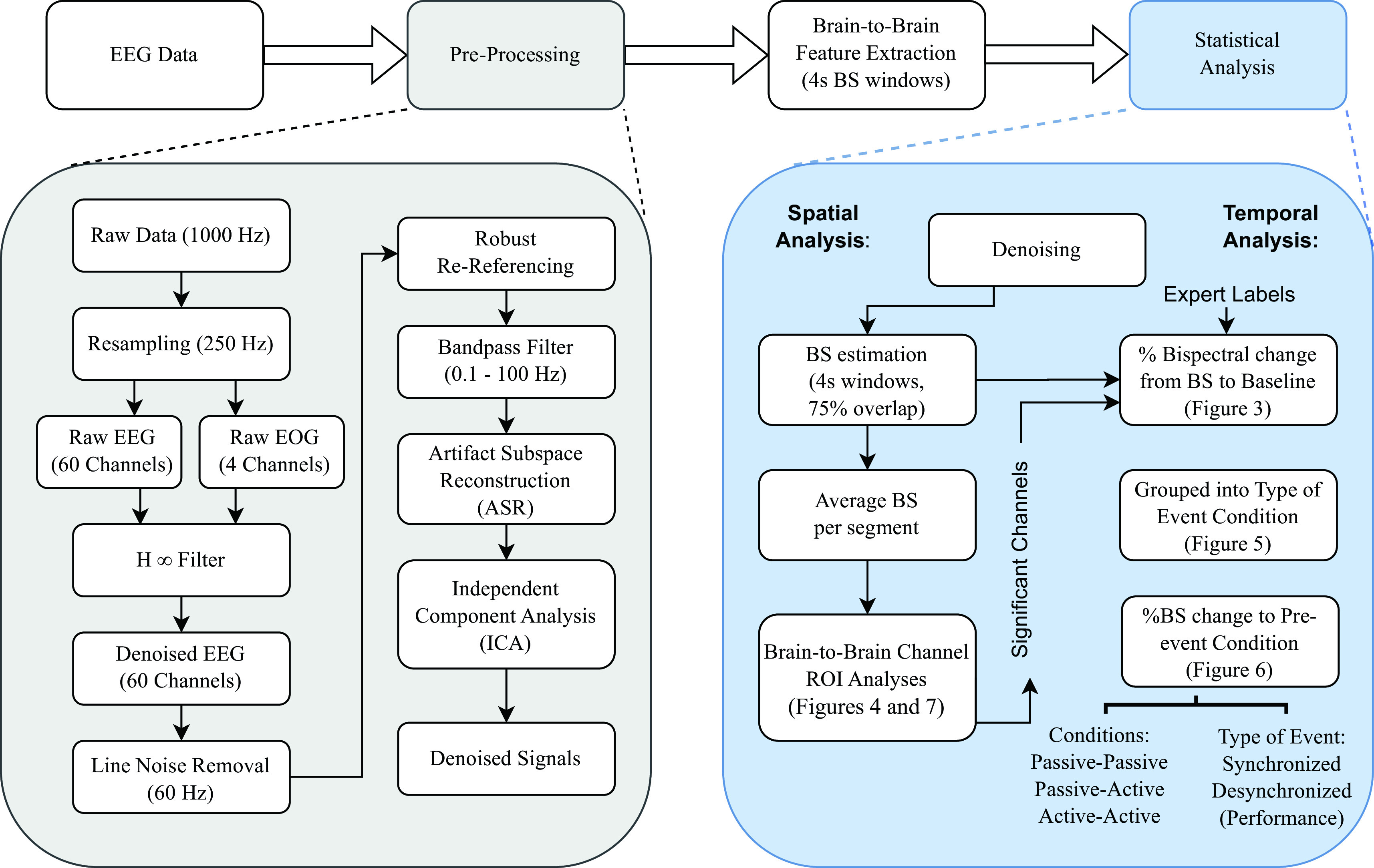
Signal processing methodology flowchart divided into four main steps: (1) electroencephalographic (EEG) data acquisition, (2) pre-processing and denoising, (3) brain-to-brain feature extraction, and (4) statistical analysis. Each step is described in detail in the Methods section.

EEG signals were acquired at 1000 Hz and resampled to 250 Hz to reduce computational cost in subsequent calculations. Signals were bandpass filtered from 0.1 to 100 Hz using a 4 ^th^ order Butterworth filter to remove unwanted noise. The PREP pipeline from the EEGLAB package ( https://sccn.ucsd.edu/eeglab/download.php) was used as the initial step to clean the data. ^60^ This procedure ensures the removal of power line noise, as well as a “true” average reference of the signals. EOG artifacts were removed from the EEG signals using an adaptive noise cancelling (ANC) framework, known as $H^{\infty }$ filter. ^61^ Raw EOG signals were used as input in the $H^{\infty }$ filter with parameters *γ* = 1.15 and *q* = 1 *e* ^−10^ for removal of eye blinks, eye motions, amplitude drifts and recording biases simultaneously. The obtained signals were further processed using the artifact subspace reconstruction algorithm (ASR) included in the EEGLAB package. ^62^ The ASR algorithm calculates the standard deviation of a “clean” portion of the signals in the principal component analysis (PCA) subspace, and reconstructs artifacts with standard deviations as *κ* times higher than in the clean portion. Here, a value of *κ* = 15 was chosen to remove remnants of eye movement and muscle artifacts. According to, ^63^ *κ* values between 10-20 are recommended to reduce artifacts and at the same time preserve the content of EEG signals. As a final step, independent component analysis (ICA) was performed on the data and suspicious components (eye, muscle, electrode popping) were removed before projecting back the signals. A graphical representation of the pre-processing steps is presented in Figure 2, as well as feature extraction and subsequent signals analysis.

### Brain-to-brain feature extraction

The improvisational nature of the performance allowed for the examination of the musical communication between the musicians as the piece progressed. At times, they built from the theme established, returned to the main theme, or proposed new ideas. With the annotations from three independent raters, we clustered sections of the performance where the participants performed synchronously or not as synchronized performance (SP), and desynchronized performance (DP). SP included moments of in-time synchronized execution, as well as improvisation and interactions under the same underlying pulse; while DP reflected moments where musicians traded material, though not aligned to the same underlying pulse, and with no coordination (as well as deviation from the current theme). A temporal (across-time) analysis was performed to observe neural synchronization in those moments of SP and DP; and these times were evaluated across three participant conditions: passive-passive, when no musicians in a dyad were performing; passive-active, when one musician in a dyad was performing; or active-active, when the two musicians in a dyad were performing. For the sake of the ecological validity approach, rather than manipulating experimental conditions (e.g. rest vs improvisation), we observed brain synchrony across SP and DP, and the participant conditions posed by the exquisite corpse approach.

The quantitative measurement of brain-to-brain communication was achieved by calculating the bispectrum between musician dyads of EEG data obtained during improvised musical performance at different stages of interactive performance. As referred to in previous works, higher bispectrum magnitudes at given pairs of frequencies reflect non-random interactions, phase coupling, ^19^ and non-linear multi-frequency interactions, ^26^ which have been observed as traces of inter-brain synchrony during teamwork interactions. ^19^^,^^24^

The denoised EEG signals were used to estimate the bispectrum between all possible channel combinations, for all participant pairs, trials and segments. Bispectrum was estimated across the EEG recordings using four-second windows with 75 *%* (one-second) overlap. The bispectrum at each time window was estimated using Equation 1:

$$B{\left(f_{i},f_{j}\right)}_{t,s,P_{\mathrm{ab}}}=\left|\sum_{l=1}^{L}X_{l}\left(f_{i}\right)X_{l}\left(f_{j}\right)X_{l}^{*}\left(f_{i}+f_{j}\right)\right|,$$ (1)

where *X _l_* ( *f _i_*) and *X _l_* ( *f _j_*) represents the Fourier transform of window *l* at frequencies *f _i_* and *f _j_* respectively, and *L* is the total number of windows. Subscripts *t* and *s* are the trial and segment where bispectrum is calculated for participants *a* and *b*, on two different EEG channels. The term $X_{l}^{*}\left(f_{i}+f_{j}\right)$ represents the conjugate of the Fourier transform of the sum of frequencies *f _i_* and *f _j._* ^64^ Using this method, bispectrum was estimated for all *f _i_* = *f _j_*, in 50 frequency bins between 1-50 Hz.

Bispectrum was estimated at 60 ^2^ EEG channel combinations between pairs of participants for all segments, and trials. A bispectral representation of a segment was obtained averaging all four-second windows in each segment, for each frequency bin (1-50 Hz). Bispectral representations were normalized to the bispectral representations of the same channel combinations during pre-trial EO task using Equation 2. Pre-trial EO was treated as rest condition, where participants did not communicate with each other.

$${\mathrm{BS}}_{N}=\frac{{\mathrm{BS}}_{\mathrm{Seg}}-{\mathrm{BS}}_{\mathrm{EO}}}{{\mathrm{BS}}_{\mathrm{EO}}},$$ (2)

where *BS _N_* is the normalized bispectrum, *BS _Seg_* is the average bispectral representation during a segment and *BS _EO_* is the bispectral representation during the EO task at the same channel combination. Normalized bispectrum representations were obtained using Equation 2 for all segments, trials, channel combinations and participant pairs. By applying this normalization, positive values of *BS _N_* for a specific channel combination represent higher temporal synchronization between specific participant pairs during performance when compared to Rest state (pre-trial EO).

Bispectral values for five frequency bands were obtained as the average of the normalized bispectral representation in the following frequency ranges: delta (1-4 Hz), theta (4-7 Hz), alpha (8-12 Hz), beta (13-29 Hz) and gamma (30-50 Hz).

A temporal bispectrum series was also estimated, using sliding overlapping windows of four-seconds (75 *%*). At each window, the temporal bispectrum values were obtained as the average normalized bispectral representation ( Equation 2) at each frequency band, thus obtaining a temporal representation of the EEG signals’ synchronization between musicians. These temporal bispectrum values were estimated for all windows using the channel combinations which were found to be significant in the implemented statistical analysis. The analysis is described in detail in the statistical analysis subsection.

### Statistical analysis

Right-tailed Wilcoxon signed rank tests were used to evaluate statistically significant differences between the average bispectrum at different frequency bands for all channel combinations. Average bispectral representations during rest and specific segments were compared.

This procedure ensures the discovery of only those channel combinations with significantly higher bispectrum at a specific segment and for a given frequency band as compared to rest. At each Segment, 60 ^2^ tests were performed ( *p* < 0.05, corrected for multiple comparisons via Bonferroni correction). Statistical tests were performed for all trials (3), segments (3), participant pairs (3) and frequency bands (5), for a total of 486, 000 tests. A different amount of samples was used for each frequency band, due to bandwidth difference; 16 for delta and theta, 20 for alpha, 68 for beta and 82 for gamma.

Through this procedure, the identified channel combinations were used as representative traces of brain-to-brain communication during musical improvisation. To further explore the behaviour of such traces, temporal and spatial analyses were implemented.

### Temporal analysis

The temporal analysis of bispectrum was implemented in representative bispectrum traces to observe its dynamics under different conditions during the performance. The bispectrum traces used in this analysis were those of the most significant channel combination (in the gamma band as described in the Results section) at the third segment of each trial, for each participant pair.

The bispectrum analysis was divided into two groups of events of naturally occurring experimental conditions: SP and DP, as labelled by the annotators in the audience. For each event, a two-minutes representative bispectrum trace in a time period (-60 to 60 s) was obtained per dyad. For each specific event, the time of the annotation was considered as the 0 s mark. To observe relative differences between SP and DP, the bispectrum traces were baseline corrected at each event for both groups. To obtain baseline corrected traces, average bispectrum in the (-60 to 0 s) period was obtained and substracted from each two-minute bispectrum trace.

Baseline corrected bispectrum traces were obtained for all events, trials and participant pairs, and were grouped and compared between the two groups. Wilcoxon signed rank tests were used to find statistically significant differences ( *p* < 0.05) at every (-60 to 60 s) time point, between SP and DP at each performance condition. This analysis was implemented independently for events in the passive-passive, passive-active and active-active performance.

### Spatial analysis

Spatial analysis was implemented to identify regions of interest (ROIs) involved in musical performance. The selected ROIs group spatially close electrodes in 13 regions: anterior frontal (AF), left fronto-central (LFC), midline fronto-central (MFC), right fronto-central (RFC), left centro-parietal (LCP), midline centro-parietal (MCP), right centro-parietal (RCP), left parieto-occipital (LPO), middle parieto-occipital (MPO), right parieto-occipital (RPO), left temporal (LT), right temporal (RT) and occipital (O). ^65^ Figure 7 shows the location for the 13 ROIs within the scalp map. The significant channel combinations identified through the statistical analysis at every segment and trial were grouped for the different performance conditions: passive-passive, passive-active and active-active.

## Results

**Figure 3.**
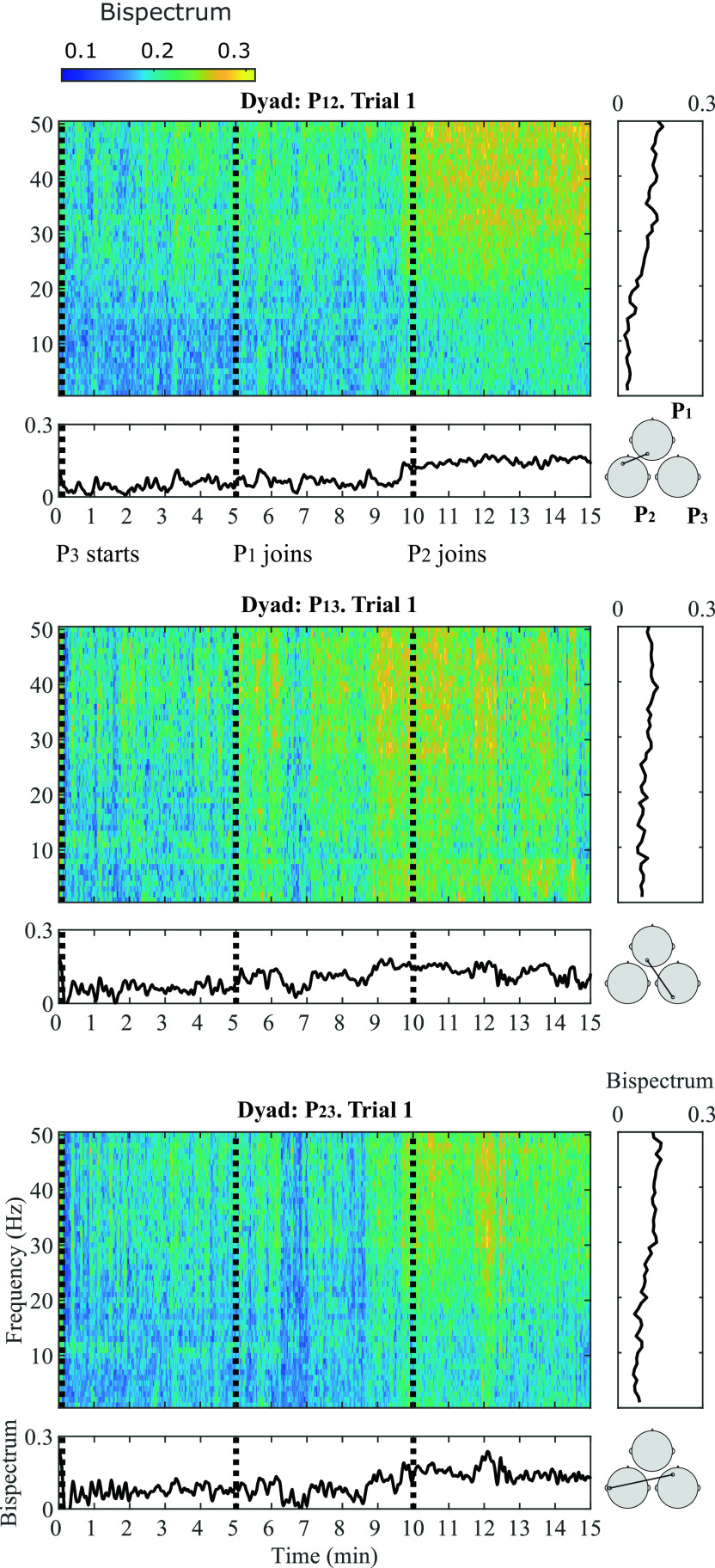
Bispectral estimations in frequency and time domain during Trial 1 for all participant pairs: P _12_ (top), P _13_ (middle) and P _23_ (bottom). For each participant pair, four insets are provided: (1) Average bispectrum in gamma band across 15 minutes of musical improvisation (bottom left); (2) Bispectrogram (1-50 Hz) across 15 minutes at (3) a representative significant channel combination in the gamma band (bottom right); (4) Average bispectrum (1-50 Hz) across the 15 minutes of performance (top right).

A general representation of the bispectral dynamics between pairs of participants during Trial 1 in shown in Figure 3. Here, normalized bispectrum is presented for the three participant pairs (P _12_, P _13_ and P _23_) in separate insets. In each inset, four plots are shown: a bispectrogram (top left), the average bispectrum at each time window in the gamma band (bottom left), the average bispectrum at each frequency bin (top right) and the most significant channel combination found at gamma band, between each dyad (bottom right).

The bispectrogram shows positive values from 0-0.3, which means that bispectrum values were up to 30 *%* higher during musical performance than during rest. ^93^ From the bispectrum representation in frequency it can be observed that in average, higher frequencies show the highest values. This particular behaviour is more evident for P _12_ and P _23_. The temporal dynamics of the bispectrum shows oscillations at different moments of the Trial, which correspond to fluctuations in EEG signals synchronization between participants. Across all participant pairs, the average bispectrum in the gamma band tends to increase from the initial segments to the latter ones, where more musicians are performing together. The regions where the highest significant synchronization was found include temporo-occipital (P _12_), occipito-occipital (P _13_) and temporo-frontal (P _23_) connections.

**Figure 4.**
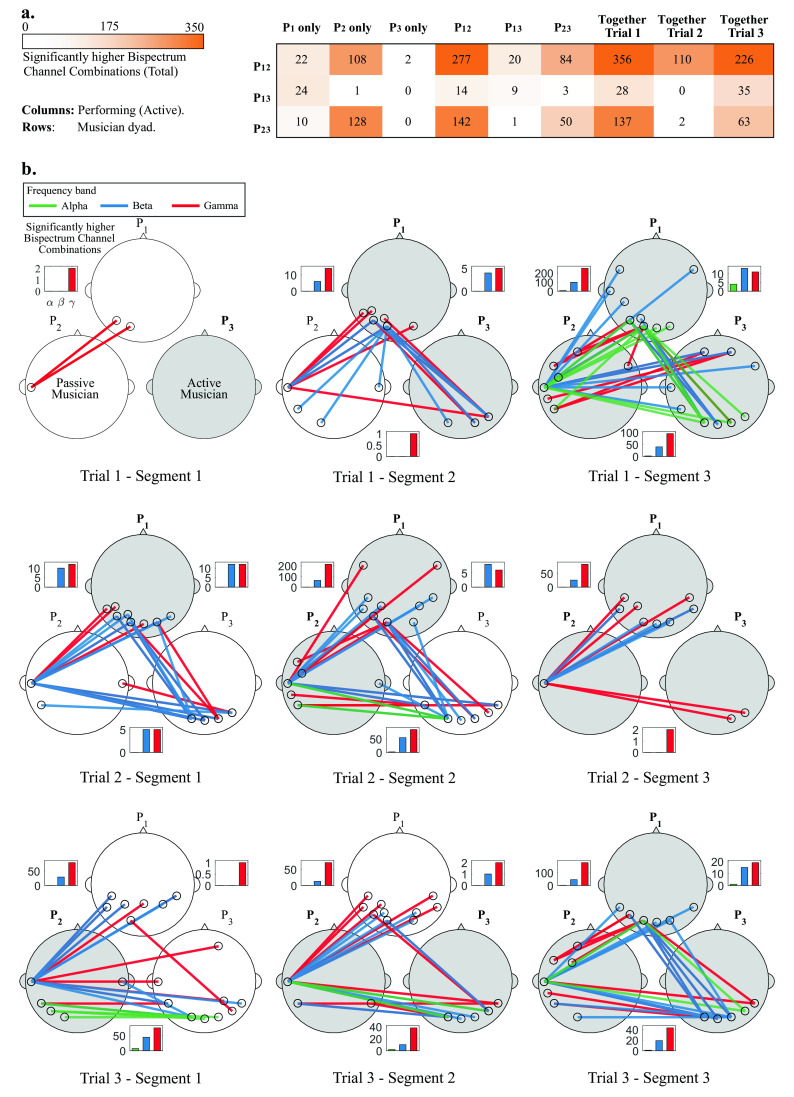
a) Total significant bispectrum channel combinations between musician dyads, across all frequency bands when one, two and three musicians perform together. b) Most significant channel combinations (up to 5) during all trials (rows) and segments (columns), for all dyads (P _12_, P _13_ and P _23_) and frequency bands (alpha, beta and gamma). Lines represent specific channel combinations with significantly higher bispectrum during improvisation than in rest condition ( *p* < 0.05) ^*^. White and gray heads show the passive and active musicians, respectively. Bars insets show the total significant channel combinations for all participant pairs for alpha, beta and gamma bands at each segment. ^*^Statistical tests were corrected for multiple comparisons via Bonferroni correction.

As Figure 1 shows, at Trial 1, P _3_ starts performing. At Segment 1, participants P _1_ and P _2_ were listening to P _3_ perform; and the bispectrum of the dyad P _12_ is lowest. The bispectrum trace of dyad P _13_ also seems low at Segment 1. The stronger bispectrum is observed for dyad P _23_. At Segment 2, P _1_ joins the play and an increase in bispectrum is observed for P _13_, as both participants are performing together. The bispectral trace of P _12_ and P _23_ show slightly higher values towards the end of Segment 2. By Segment 3, P _2_ joins the other two participants and all are improvising together. Bispectrum increases at all participant pairs are observed during this final Segment, reflecting higher EEG synchronization, when compared to segments where participants are not actively interacting in the performance. These observations were tested statistically for all channel combinations in Figure 4.

### Statistical analysis

The procedure of the statistical tests presented in the Statistical analysis subsection was implemented for all Segments, Trials, frequency bands and participant pairs. No significant channel combinations were found for the delta and theta band for any segment. Most statistically significant channel combinations were found for the beta and gamma bands, and a few in the alpha band.

Figure 4(a) shows the total significant channel combinations (between all dyads) when different musicians were performing together, and Figure 4(b) shows a topographical representation of the statistical analyses.

In Figure 4(a), the dyads P _12_ and P _23_ show consistently more significant inter-brain synchronized channels that for the P _13_ dyad. The three dyads showed few synchronized channels when musician P _3_ performed alone, and when P _1_ performed together with P _3_.

The most significant channel combinations (visualizing the top 5 channel pairs) for the alpha, beta and gamma bands are shown for each participant pair. Bar graphs show the amount of significant channel combinations at each frequency band, and dyad. At each specific segment, passive and active musicians are shown as white and gray heads, respectively. Topographical representations are presented for all trials and segments, therefore the first row of Figure 4 corresponds to the data shown in Figure 3.

In Trial 1 (top row of Figure 4(b)) and Segment 1, P _3_ starts performing, and only a few significant channel combinations were found for dyad P _12_ in the gamma band. In Segment 2, P _1_ joins and more channel combinations are shown in the beta and gamma bands for dyad P _12_ and P _13_, who are performing. In Segment 3, when all musicians are performing, an increase in the amount of significant channel combinations is observed for all participant pairs. In this last Segment, a few channel combinations were observed in the alpha band.

In Trial 2 (middle row of Figure 4(b)), in Segment 1, P _1_ starts performing. A few channel combinations were found to be significant for all participants in the beta and gamma bands. In Segment 2, P _2_ joins and an increase in the amount of significant channel combinations is observed for dyads P _12_ and P _23_). In the Segment 3, P _3_ joins and a decrease in the amount of significant channel combinations is observed across all participants).

In Trial 3 (bottom row of Figure 4(b)), P _2_ starts performing, and significant channel combinations for P _12_ and P _23_ are observed for beta and gamma, and only one for P _13_. In Segment 2, P _3_ joins and a similar connection pattern is observed between participants at Segment 1. In Segment 3, P _1_ joins and a considerable increase in significant channel combinations is observed for both P _12_ and P _23_, while those for P _23_ remain similar.

Some general patterns were observed through this analysis. It was observed that the amount of significant channel combinations increased as more musicians joined the performance, which can be observed in Figure 4(a). Dyad P _13_ showed less amount of significant channel combinations throughout the experiment, at different segments and trials 4 (a). Also, in all segments, the amount of significant channel combinations was higher for the gamma band than for beta or alpha bands. Finally, the most common interconnected regions across segments and trials are those involving the temporal, occipital and parietal regions.

### Temporal analysis

**Figure 5.**
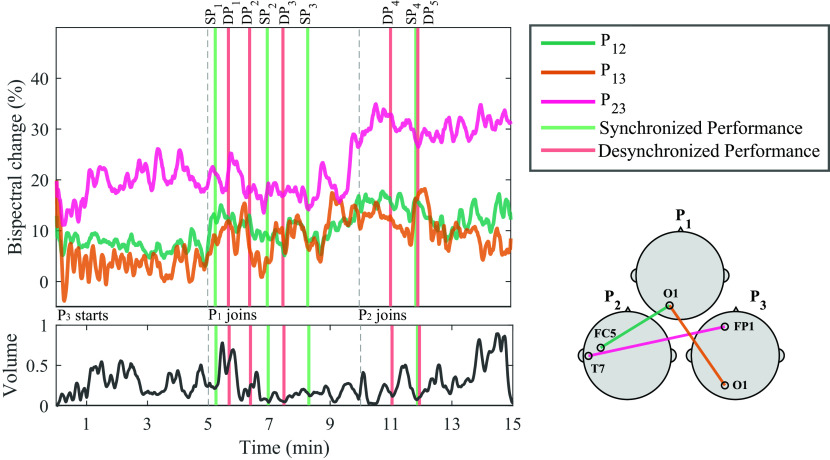
Bispectrum temporal dynamics at the most significant channel combination per participant pair in the gamma band, and normalized volume intensity (unitless) of the audio recorded during the performance of Trial 1. Vertical dashed lines represent the times when a new musician joined the performance. Vertical bars represent time of synchronized performance (SP) or desynchronized performance (DP) events, as labelled by experts. A representation of the selected channels for each dyad is shown in the bottom right corner. The annotations from the events are shown in Table 1.

Figure 5 shows the normalized bispectrum trace for the 15 minutes of Trial 1, using the significant channel combinations described in the temporal analysis subsection. The vertical dashed lines mark the division between segments, and bars are used to visualize the moments during the performance when the experts identified either an SP or DP event. The volume of the recorded audio file from the performance is shown below the bispectrum traces. The individual events shown in Figure 5 are described in Table 1. The corresponding Figures and Tables for Trials 2 and 3 are presented in the *Extended data,* in Figures S1 and S2, and Tables S1 and S2, respectively. Representative SP and DP events from Trials 1-3 are presented in Videoclips S1-S3 in the *Extended data.* ^93^

**Figure 6.**
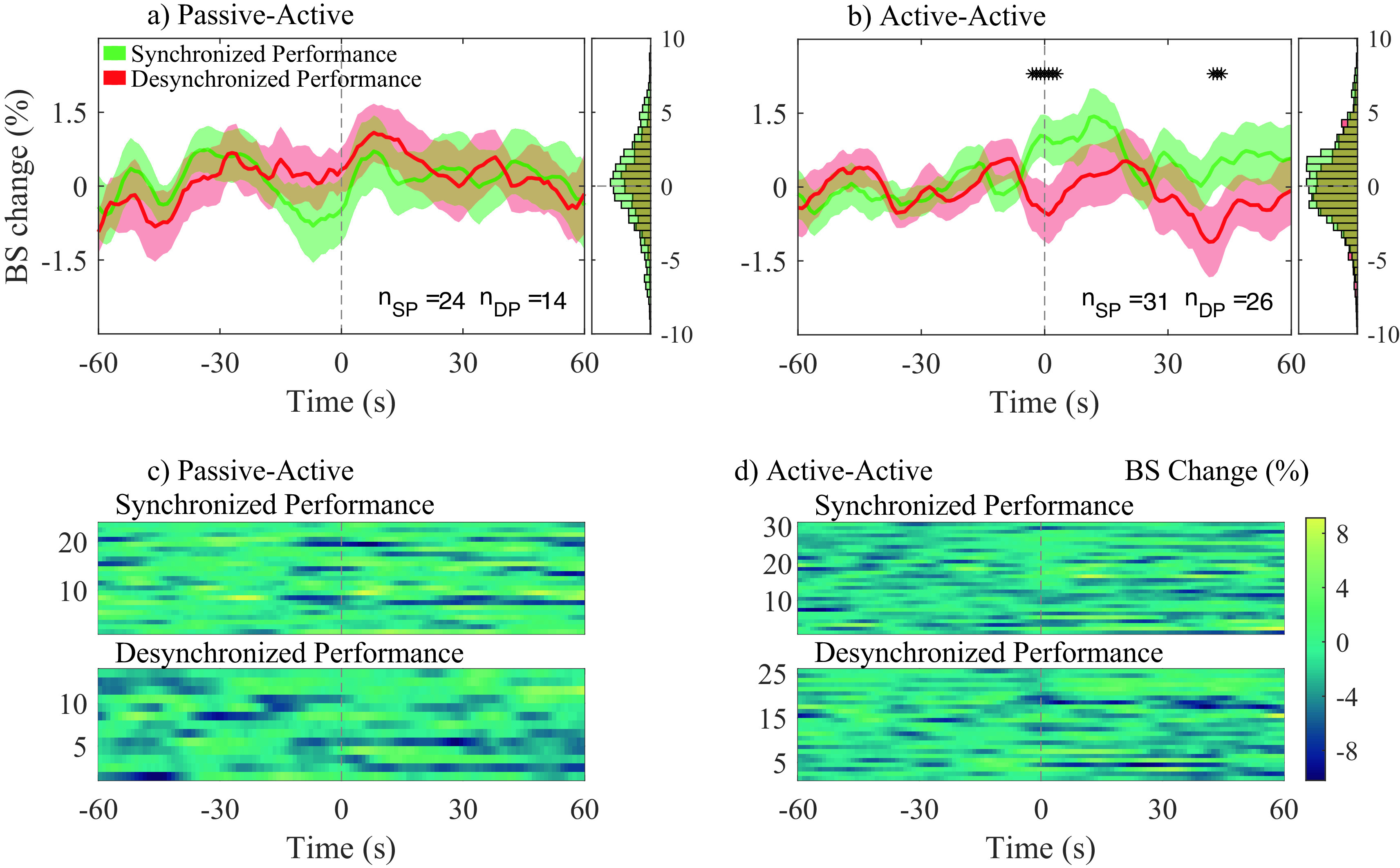
Average bispectrum (BS) change (%) across all segments and participant pairs at their most significant channel combination in gamma band, for both synchronized performance (SP) and desynchronized performance (DP) events. BS change (%) was obtained applying baseline correction on each trace by using the 60s previous to each event. Average BS changes (%), and histograms (distribution of all traces) are shown for passive-active (a) and active-active (b) conditions. The amount of averaged traces at a) and b), respectively, are: 24 and 31 during SP (n *_SP_*); 14 and 26 during DP (n *_DP_*). Individual BS (%) changes for all n *_SP_* and n *_DP_* events are presented in c) and d) for the passive-active and active-active conditions, respectively.

Figure 6(a) and (b) show the average bispectrum change across participant pairs for the passive-active and active-active conditions, respectively, for both SP and DP. The 0 seconds vertical dotted line in Figure 6 indicates the start of the event: either SP or DP. The amount of averaged traces for each condition were, 24 and 31 for SP; and 14 and 26 for DP; for the conditions passive-active and active-active, respectively. Figures c) and d) show every individual trace analyzed in a) and b), respectively.

No statistical significance was observed between SP and DP in the passive-active condition. Bispectrum change was significantly higher during SP than DP in the active-active condition, slightly before the onset of annotated events (− 3 s), as well as 40 s after the onset.

### Spatial analysis

**Figure 7.**
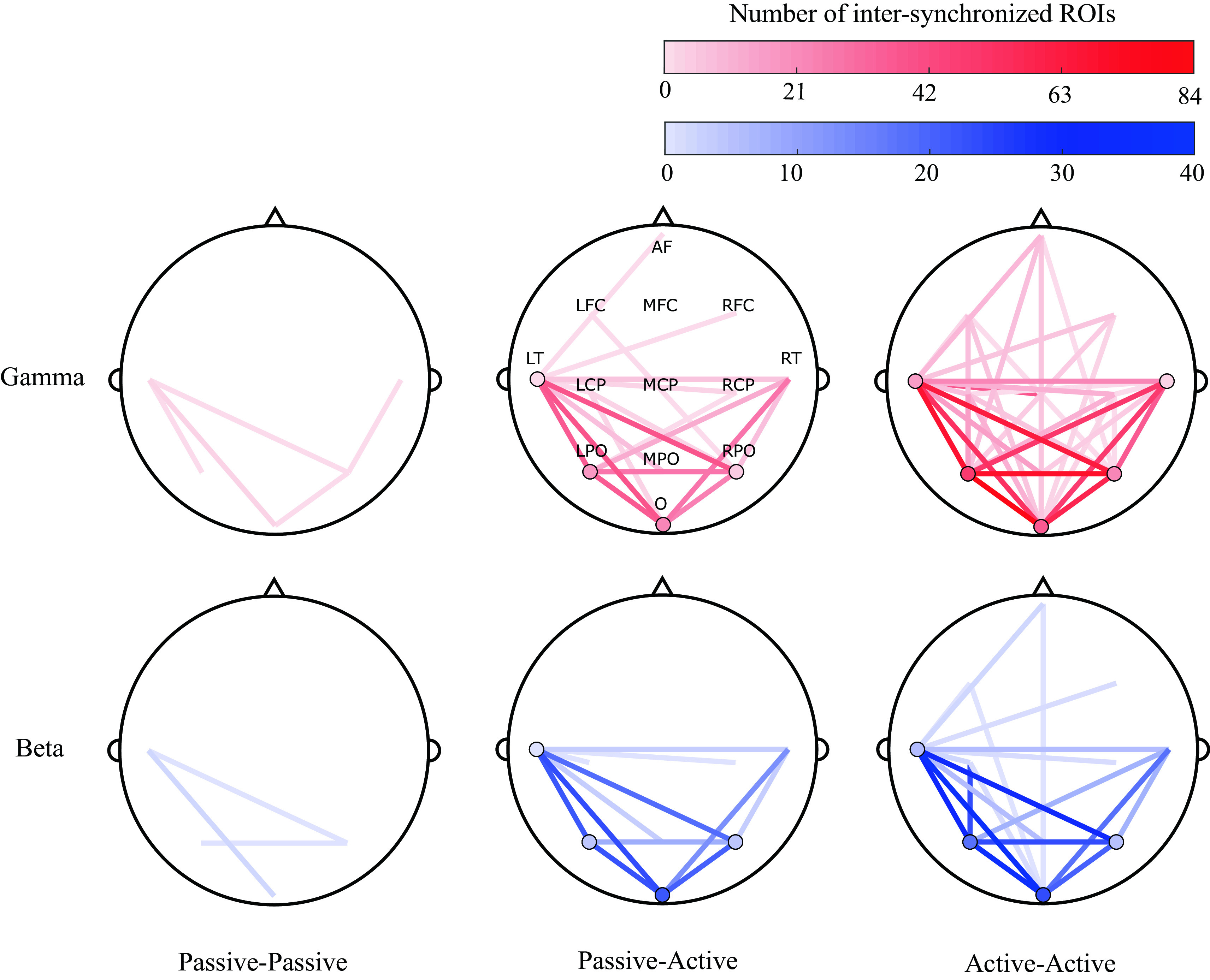
Topographical representations of between-participants inter-synchronization of 13 regions of interest (ROIs) (anterior frontal, left fronto-central, midline fronto-central, right fronto-central, left centro-parietal, midline centro-parietal, right centro-parietal, left parieto-occipital, middle parieto-occipital, right parieto-occipital, left temporal, right temporal and occipital) across all dyads (3) and trials (3), in passive-passive (left), passive-active (middle) and active-active (right) conditions, in the gamma (top) and beta (bottom) bands. Shading represents the degree of inter-synchronization within the same (circles) and different (lines) ROIs.

A topographical visualization of the most significant scalp ROIs for participant pair conditions (summarizing the findings of Figure 4), is shown in Figure 7. Visualization maps were plotted to represent the degree of synchronization between and within the evaluated ROIs for all conditions (passive-passive, passive-active and active-active), dyads (3) and trials (3). Figure 7 show this representation in the gamma and beta bands. It can be observed that the most synchronized ROIs are in the active-active condition, while less are observed in the Passive-Active condition and the lesser during passive-passive condition.

### Summary of main findings

Brain synchrony (as assessed via the bispectrum) increases when more musicians are performing. This is most notorious in the higher frequency bands (beta and gamma).

The number of inter-connected channels and ROIs increase when there are more musicians performing. This happened mainly across temporal, occipital and parietal regions, related to auditory, visual and spatial processing.

Changes in brain synchrony (bispectrum) allowed to identify different performing states across musicians during the improvisation: Bispectrum was higher during Synchronized Performance and lower during Desynchronized Performance.

## Discussion

The bispectrum analysis allowed us to obtain a quantitative representation of brain-to-brain communication, by analyzing the temporal synchronization strength (i.e. bispectrum) of EEG signals between musicians during a free jazz improvisation performance. In such performances, musicians continuously engage in a dynamic communication formed by perception, evaluation and action. The presented methods were applied to observe the synchronized interactions of neural activity at different stages of the performance, different recording sites and under five frequency bands.

Following our proposed methods, a bispectral representation in the time and frequency domain were obtained for all pairs of possible combinations of the assessed variables (segment, trial, frequency band, and channel). Statistical analysis revealed that the most significant synchronization between EEG signals of paired musicians were found in high frequency bands: beta and gamma, as shown in Figure 4. Also, in the same analysis, it was noted that most of the significant neural synchronization links were formed between the occipital, parietal and left temporal regions. Such results were used to assess the most frequent connections between ROIs across pairs of musicians at different performance conditions (See Figure 7).

Although musicians exhibit differences in their brain activity due to individual preferences, domain-specific memory for previously encountered auditory-motor patterns, ^36^^,^^66^ as well as the nature of their instruments (e.g. drummers use more spatial and visual processing), common patterns were observed. In this study, the most synchronized ROIs between musicians were found at left temporal, and bilateral parietal and occipital sites (LT, LPO, O, RPO and RT), with increased synchronization in the beta and gamma bands.

These results have further implications for cross-modal plasticity due to musical training, between individuals. The posterior coupling between musicians can be strengthened through extensive training. ^67^^,^^68^ Such processes are present when musicians rhythmically engage in a collaborative, creative work. Two processes give rise to this dynamical sensorimotor integration: motor commands, and sensory predictions that provide feedback regarding the given command. ^68^^–^^70^ This feedback loop often informs individuals of errors or mismatches between predicted and real sensory feedback, which results in the reconfiguration of this perception-action cycle. ^70^^,^^71^ However, this cycle is not restricted to self-generated action. An increasing body of research suggests that in musical contexts, musicians are able to form multiple action representations, performing real-time integration of perceptual stimuli, motor commands, and outcome predictions for one-self and others. ^68^ This complex, moment-to-moment processing within the perception-action cycle, informed by internal forward models, may be the foundation of inter-personal synchrony in creative, musical contexts. ^68^

Neural synchronization fMRI studies of resting state in musicians have found increased functional connectivity between the auditory and motor cortices within an individual’s brain ^72^ and in default mode network and executive control network. ^73^ fMRI studies have shown long term induced plasticity ^74^ in trained musicians when compared to non-musicians. Improvising jazz musicians experience weaker connectivity in pre-frontal areas during musical improvisation, compared to performing pre-learned segments. ^75^ Studies in the literature resembling our findings regarding gamma band activity in music related processes have been reported; expert musicians exhibit neural synchronization between multiple cortical areas in the gamma band ^76^ and left hemispheric gamma synchrony while listening to music ^77^ while such patterns are not observed for non-musicians. Inter-brain synchronization in the theta/alpha amplitudes between temporal and lateral-parietal regions has also been described during speech-rhythm coordination in. ^78^ The results obtained from the statistical and the ROI analysis suggest that beta and gamma synchronization is present during the performance of higher cognitive tasks that need a dynamic binding of information, such as an improvised collaborative musical performance. In our case study, the presence of higher synchronization between temporal, parietal and occipital sites during improvised musical performance suggests the establishment of functional inter-connections between musicians which reflect shared multi-sensory (visual, auditory, and spatial, respectively) processing, integration, and communication. ^28^ Auditory and visual cues from co-performers have been reported to relate to the strength of inter-musician coordination during musical improvisation. ^79^

These results show evidence for an inter-musician perception-action cycle, where there is a circular, feedback-based, hierarchical method of information-processing conducted by the interplay between both posterior (i.e. sensory input) and anterior (i.e. motor, executive output) regions of the cortex. ^71^ In this experiment, inter-brain bispectrum analysis showed synchronicity in sensory areas. Cross-modal plasticity, and reinforcement of intra-brain coupling of posterior and anterior areas, has been shown to be enhanced by musical training. ^68^ Experience in joint performance leads to fine-tuning of the internal forward model representation that allows for the prediction of observed or listened actions from fellow musicians with high temporal resolution. Our results suggest that coupling in posterior and temporal regions is associated with such predictions of the actions from other members of the performing group. The musicians generate predictions both about when and what their peers’ new musical idea will occur. Musicians with experience performing together may in fact learn which succession of tones are likely to occur, stemming from regularity from previous performances. This complex, moment-to-moment processing within the perception-action cycle, informed by internal forward models, may be the foundation of inter-personal synchrony in creative, musical contexts.

Musician P _1_ and P _3_ performed together for the first time in this experiment, while musician P _1_ and P _2_ performed regularly together prior to this study. Thus, it is likely that P _1_ and P _2_ had developed strong internal forward models of each other that enabled them to predict and respond to recognized sequences between them, as shown in Figure 4(a). Musician P _3_ had the lesser prior musical collaboration with the other musicians. This difference in familiarity background supports the finding of a smaller number of synchronized channels between P _3_ and the other musicians throughout the three trials of the performance.

Across all trials of the present study, significant bispectrum synchronization was found in posterior (e.g., parietal, temporal and occipital) regions that are involved in the processing of sensory input and are important in interpreting sensory feedback from the external environment. Because musical improvisation is founded in nuanced, interpersonal exchange of motor commands that are generated based on constantly evolving sensory input, these findings support the notion that this musical, creative synchrony between participants is highly dependent on the sensory, perceptual inputs they are receiving from each other, and their surroundings. The output in this cycle (i.e. action) would be represented by activation of anterior (e.g. frontal) regions. In Figure 7, connections involving anterior regions are more present during active-active interactions, when both musicians are performing (producing an action) together, while a predictive component can also be observed in Figure 6, where a significant positive bispectrum change was observed across all analyzed SP events approximately three seconds before the onset of the labelled events. Both components were not present during passive-active and passive-passive interactions, in which action and anticipation are not as needed due to the nature of such conditions.

Another key variable to address in our study is the temporal dynamics of the bispectrum. In Figure 5, the temporal bispectrum dynamics show a consistent increasing trend, as more musicians joined the performance. Towards the final segments of the performance, there were more’musical voices’ interacting, increasing the complexity of the piece, as well as the stimulation, perception, and engagement. This increase in bispectrum was also observed in Trial 3 for P _12_ and P _13_, but not for P _23_, which presented a decreasing trend (See Figures S1 and S2, *Extended data* ^93^). Also, in Trial 2, a general bispectrum decrease was observed for all dyads, with a sudden increase at the end of the first segment, where a new musician joined the performance. As mentioned in the Experimental design subsection, P _3_ is a classical trained musician, while P _1_ and P _2_ are professional jazz musicians. It is also important to mention that P _1_ and P _2_ often perform together, while P _3_ is not an acquaintance of them. Brain-to-brain synchrony has been studied between dyads under different social contexts, such as between romantic couples and strangers ^21^^,^^22^ and it has been reported that higher neural coupling relate to the degree of social connectedness and mutual pro-sociality. It has also been noted from recent musical improvisation studies that the familiarity between musicians predicts stronger coordination of intentions during the performance. ^80^ Increased neural synchrony between two participating individuals may indicate mutual, efficient, and effective social interaction ^81^ and can be modulated by the degree to which the participating individuals feel socially connected, the activity they are engaging in, and the interaction setting. ^21^^,^^81^^–^^83^ An interpretation of the aforementioned temporal bispectrum changes is that during bispectrum decreases, participants were not communicating effectively, and the “closeness” between each other had an important role in this communication. It can be observed from the bars in Figure 4 that a lower amount of significant channel combinations were found at the dyads where the unacquainted P _3_ is present, whereas a higher amount of significant channel combinations are found between the more acquainted musicians P _1_ and P _2_. This is also evident in Figure 4(a), where the highest amount of significant channels is presented between P _1_ and P _2_, and lower combinations are significant when P _3_ is involved.

By analyzing the fluctuations of bispectrum change relative to the stimuli type and onset, differences in bispectrum dynamics were observed whether musicians were performing in synchronization or not. This analysis revealed on average higher bispectrum during synchronized performance when compared to desynchronized performance. Higher inter-brain synchrony has been reported in participants performing cooperative tasks and lower synchrony when performing competitive tasks. ^15^ Based on the results of this study, higher bispectrum was observed between participants while performing in synchrony, which might be a reflection of cooperative intention. On the other hand, lower bispectrum values during desynchronized performance might be indicative of a competitive behaviour (e.g. changing the current theme or proposing a new idea). A review on this topic is presented in, ^16^ however it is noted that most papers in this regard are based on an experimental design under controlled laboratory settings. In our study, a real-world scenario is presented, therefore it is best suited to study these types of interactions. An interesting note on this regard is that in a musical improvisation, alignment and misalignment between musicians are both needed to contribute a new perspective on an established theme, ^54^ and continuously propose new musical paths in the piece. Moreover, the observed brain synchrony dynamics are associated to the spontaneous decisions and interactions of the musicians during an unconstrained free jazz improvisation, which does not facilitate the temporal prediction that a steady pulse would cause in the musicians. ^30^

Our results suggest that bispectrum was able to detect relevant temporal and spatial information about musician’s interactions during the performance. Therefore, the proposed method could be used to track the degree of synchronized interactions and can be applied to different contexts. Some of the applications and desired outcomes of research in this field is the development of neural biomarkers that measure in real-time the quality or the strength of shared cognitive states such as: brain-to-brain communication, shared attention, message conveying, and high engagement during human interactions. Two possible applications are the use of such methods to track changes in social interactions in patients suffering from communication disorders, and to enhance learning in educational settings.

While the social nature of individuals has been recognized and acknowledged as foundational to human interaction, research regarding the neural inter-brain basis of these interactions naturalistic social settings has only just begun in its investigation. ^83^ Hyperscanning applied to social interactions opens the possibilities to study and enhance social exchanges. ^15^ In a recent study, large groups of museum-goers participating in face-to-face pairs in an artistic neurofeedback installation were found to exhibit higher levels of inter-brain synchronization in low alpha and beta band frequencies, correlated with the pair’s empathy, social closeness, engagement, joint action and eye-contact. ^84^ Observing brain-to-brain synchronization during naturalistic exchanges could not only aide in developing a more comprehensive understanding of its neural underpinnings, but also could shed light on various communication disabilities. ^81^

A bispectral analysis to observe brain-to-brain synchronization during social interactions could be used in the educational field to increase teacher-learner synchronization to enhance learning outcomes and experiences. ^85^ A study examining the effects of brain-to-brain synchronization within a classroom setting was performed on a group of four science students and a teacher. In such study, alpha-band synchrony between students significantly predicted subsequent performance (i.e., memory retention) on both immediate and delayed post-tests. ^86^ Inter-brain synchronization in prefrontal and superior temporal cortices between instructor-learner dyads has been shown to increase when the instructor engages with the learner through guiding questions and hints. ^87^ Such results are also consistent with previous research on the synchrony between speakers and listeners. ^86^^,^^88^^,^^89^ Brain-to-brain synchronization in a naturalistic musical performance provides a window to assess the perception-action and communicative cognitive processes required during musical improvisation ^45^; and coupled with instructor-learner interactions, inter-brain synchronization metrics can inform effective pedagogical techniques.

This study faced some limitations given the logistical challenges of integrating performance and MoBI research in a public setting. First, this study has a small sample size (three professional musicians). However, this drawback can be justified by the ecological validity of our experiments, which were intended to capture the interactions of musicians during real-world improvised performance, ^29^^,^^30^ and the experimental design that included counterbalancing to allow for testing different participants in different orders. The authors believe the ecological approach and experimental methodology used herein represent a milestone in the acquisition and understanding of brain data “in action and in context”, and the development of brain-to-brain communication metrics. It is of interest of the authors to implement the presented methods in different experimental designs oriented to unveil the shared neural traces of human interactions under a variety of contexts (e.g., dance, theater, teaming, education, etc.).

Another limitation of this study is the potential lingering effects of artifacts associated to the movements needed to perform music through the (percussion and wind) instruments involved in the experiments. Such artifacts are likely related to body and facial movements, blowing, head swaying, among others, and can contaminate the EEG signals. Although we deployed well known pre-processing and de-noising methods found in the literature ^1^^,^^2^^,^^90^ and performed visual inspection of the raw and cleaned data, it is still possible that residual motion and muscle-related artifacts may still remain in the processed EEG signals, and thus these results may be taken with caution. As additional information on this note, a comparison of EEG signals’ independent components (ICs) before and after the de-noising framework implemented in this study is presented in Figures S5-S10 ( *Extended data* ^93^). On the other hand, the existence of body movements can provide insight on behavioural synchronization between musicians. ^91^ At the same time, it has been reported that movement synchrony in groups is correlated to the presence of positive emotions. ^92^ Although out of the scope of this work, because of the focus on brain signals' synchrony, movement synchrony and emotional context (across musicians and with the audience) are undoubtedly interesting under the studied scenario, and will be considered for analysis in future steps of this research.

Nevertheless, the presence of movement artifacts will be present in any ecologically valid study on musical improvisation due to the freedom of movement of the musicians. ^29^ Additionally, this performance case study offers a novel way to investigate inter-brain synchronization, in “action and in context”, during a free jazz improvisation in real-world scenarios by expert musicians.

## Conclusion

In this study, temporal synchronization of EEG signals between musicians interacting during a jazz performance was observed through a bispectral analysis. The most significant interactions were found between left temporal, bilateral occipital and parietal regions at the gamma band, which reveals a shared dynamic and synchronized processing of auditory, visual and spatial information needed during a cooperative improvised performance. The inter-brain interaction between electrodes in sensory integration areas among musicians provides evidence towards the centrality of sensory processing, ^47^ feedback, ^45^^,^^66^ and communication ^45^^,^^46^ during a collaborative musical improvisation.

A temporal analysis of the bispectrum dynamics for both synchronized and desynchronized performing allowed to observe higher bispectrum when musicians were performing in a synchronized manner, when compared to desynchronized performing. In this study, bispectrum was useful to identify differences in competitive and collaborative performance in a real world scenario such as musicians improvising a collaborative piece. Based on the presented results, the implemented bispectral analysis method is proposed to study social interactions and brain-brain communication in hyperscanning measurements.

## Data availability

### Underlying data

OSF: MOBILE EEG RECORDINGS OF MUSICAL (JAZZ) IMPROVISATION.

https://doi.org/10.17605/OSF.IO/YUEQK ^93^

This project contains the following underlying data:

• Block1_P1.mat (EEG data - Recording Block1, Participant 1).
• Block1_P2.mat (EEG data - Recording Block1, Participant 2).
• Block1_P3.mat (EEG data - Recording Block1, Participant 3).
• Block2_P1.mat (EEG data - Recording Block2, Participant 1).
• Block2_P2.mat (EEG data - Recording Block2, Participant 2).
• Block2_P3.mat (EEG data - Recording Block2, Participant 3).
• Impedances.xlsx (Impedance values of EEG electrodes from all participants, at start and end of recordings).
• Performance Notes.xlsx (Notes with times of trials, segments and relevant events during the performance).
• ZOOM0001.mp3 (Audio recording of the complete performance).
• Blaffer_Floor_1210.mp4 (Video Recording1 from the performance).
• Blaffer_Floor_1221.mp4 (Video Recording2 from the performance).

### Extended data

OSF: MOBILE EEG RECORDINGS OF MUSICAL (JAZZ) IMPROVISATION.

https://doi.org/10.17605/OSF.IO/YUEQK ^93^

This project contains the following extended data:

• Extended Data.pptx (An extended data file containing additional figures and tables from this work).

Data are available under the terms of the Creative Commons Zero “No rights reserved” data waiver (CC0 1.0 Public domain dedication).
