# Supplementary material for: Brain-to-brain communication during musical improvisation: a performance case study
Source: F1000Res. 2023 Nov 3;11:989. Originally published 2022 Sep 1. [Version 4] doi: 10.12688/f1000research.123515.4 (PMC10558998; doi:10.12688/f1000research.123515.4)
Supplement: Peer review history

# Reviewer report: Reviewer response for version 4

**DOI:** 10.5256/f1000research.158355.r220645

**Published:** 2023-11-16

**Authors:** Guido Orgs

**Affiliations:** 1 Department of Psychology, Goldsmiths, University of London, London, UK

I have no further comments to make

Is the case presented with sufficient detail to be useful for teaching or other practitioners?

Yes

Is the work clearly and accurately presented and does it cite the current literature?

Yes

If applicable, is the statistical analysis and its interpretation appropriate?

Yes

Are all the source data underlying the results available to ensure full reproducibility?

Partly

Are the conclusions drawn adequately supported by the results?

Partly

Is the background of the case’s history and progression described in sufficient detail?

Yes

Reviewer Expertise:

Dance, Neuroaesthetics, Action Observation, EEG Hyperscanning.

I confirm that I have read this submission and believe that I have an appropriate level of expertise to confirm that it is of an acceptable scientific standard.

# Reviewer report: Reviewer response for version 3

**DOI:** 10.5256/f1000research.152004.r201607

**Published:** 2023-10-06

**Authors:** Nicolas Farrugia

**Affiliations:** 1 IMT Atlantique, Brest, France

The authors here present an ecological study on brain to brain synchrony during free jazz improvisation with three musicians. They used EEG on the three musicians, with an experimental design that enables to test whether inter brain synchrony was modulated as a function of who's playing with who, and with active / passive roles in improvisation. The setup is particularly challenging to do its ecological validity, and due to noise related to movements in the EEG. After extensive preprocessing, the authors have performed a very elaborate analysis pipeline focusing on time-frequency analysis, and in particularly on bispectrum estimation. The presented results show many interesting effects related to the interplay between musicians, and are quite clearly presented and convincing. Overall, the paper is well written, the statistical analysis is sufficient and the results are convincing towards the given objectives. This kind of ecological study is really welcome, and I want to congratulate the authors for running this complex project and encourage to investigate follow up questions in future work. For the time being, I only have a few points which I think would make an overall better contribution, that are detailed below.

 Major issues :

1. As awkward as it can sometimes be to ask to add one's own reference, I think it would make sense to cite our previous study on a single case study on improvisation, also done using EEG, in a free jazz context (Farrugia et al. 2021, ref 1 below). While it's not an hyperscanning study, it s an ecological study with an audience, so it would make sense to at least cite it in the related works. Our paper investigated other questions on the subjective state of the improviser, namely flow state and subjective time perception.
2. Another interesting reference to add would be the review by Fairhurst and Dumas, ref 2 below.
3. The OSF link to the data is broken https://osf.io/yueqk so I cannot guarantee that the data is accessible currently (reference 96 in the manuscript
4. The following statement in the discussion is very speculative, and not backed by any references. *"An interesting finding from this study is that brain-to brain synchrony appears to be limited to the higher frequency bands. Although it may seem that music performance requires behavioral coordination across these longer timescales (i.e. musical phrases for example) that may involve delta and theta bands, it is possible that the jazz improvisation performed by the musicians imposed higher demands in higher frequency bands, which are also more localized. Future studies comparing jazz improvisation with other types of music performances that may emphasize longer timescales could elucidate this question."* In  [Farrugia et al. 2021], we did find modulations in the theta and beta oscillations related to subjective states of the improviser, and this was also on "free jazz" (see for example https://www.youtube.com/watch?v=ILhaZYtW8fs&t=390s ). But still, I don't find the statement about linking musical timescales and oscillations frequencies very convincing overall. I'm also not sure what the authors mean by "more localized" in this context. I would recommend removing this paragraph.
5. Authors should refrain from doing direct inferences about neural sources based on scalp electrode positions, such as " *The number of inter-connected channels and ROIs increase when there are more musicians performing. This happened mainly across temporal, occipital and parietal regions, related to auditory, visual and spatial processing*.", or " *posterior (e.g., parietal, temporal and occipital) regions that are involved in the processing of sensory input and are important in interpreting sensory feedback from the external environment*". It's ok to mention interconnected channels and ROIs, but it's impossible to say how much this is related to auditory, visual and spatial processing without doing source localization. Generally speaking, in the results and in the discussion, the authors should stick to channel positions and ROIs at the scalp level, without linking those to brain areas or brain functions.

Minor issues :

- "Towards the final segments of the performance, there were more ’musical voices’ interacting, " a space is missing before 'musical
- A very general question related to one of the limitations that is discussed. The authors do mention that the observed effects might be related to movement artifacts ; is there any statistical analysis that can be presented in this paper to at least show that such a confound is equally "strong" across conditions (e.g. between synchronized and not synchronized performances)

Is the case presented with sufficient detail to be useful for teaching or other practitioners?

Yes

Is the work clearly and accurately presented and does it cite the current literature?

Partly

If applicable, is the statistical analysis and its interpretation appropriate?

Yes

Are all the source data underlying the results available to ensure full reproducibility?

Partly

Are the conclusions drawn adequately supported by the results?

Yes

Is the background of the case’s history and progression described in sufficient detail?

Yes

Reviewer Expertise:

Auditory cognitive neuroscience, prior work on music-based rehabilitation of Parkinson's disease using ryhtmical auditory cueing, as well as musical improvisation with EEG, and music neurosciences in general.

I confirm that I have read this submission and believe that I have an appropriate level of expertise to confirm that it is of an acceptable scientific standard, however I have significant reservations, as outlined above.

## References

[1] : Reciprocity and alignment: quantifying coupling in dynamic interactions. *R Soc Open Sci* .2021;8(5) : 10.1098/rsos.210138 210138 10.1098/rsos.210138 34040790PMC8113897

[2] : Beta and Theta Oscillations Correlate With Subjective Time During Musical Improvisation in Ecological and Controlled Settings: A Single Subject Study. *Front Neurosci* .2021;15: 10.3389/fnins.2021.626723 626723 10.3389/fnins.2021.626723 34177443PMC8222590

# Response

**Published:** 2023-10-30

**Authors:** Mauricio Ramirez

**Affiliations:** University of Houston, USA

**We thank the reviewer for the time invested in reading and making such useful comments for our work. Below is a response to each of the comments (in bold letters), as well as the locations of the corrections made to the manuscript. All changes are carried out in the next version (Version 4) of the manuscript, which should be published soon.**

 Major issues: 

1. As awkward as it can sometimes be to ask to add one's own reference, I think it would make sense to cite our previous study on a single case study on improvisation, also done using EEG, in a free jazz context (Farrugia et al. 2021, ref 1 below). While it's not an hyperscanning study, it s an ecological study with an audience, so it would make sense to at least cite it in the related works. Our paper investigated other questions on the subjective state of the improviser, namely flow state and subjective time perception.
2. Another interesting reference to add would be the review by Fairhurst and Dumas, ref 2 below.

**Introduction, Brain-to-brain synchrony between musicians in free-jazz improvisation, Paragraph 2. Both references were added. As stated by the reviewer, although not using hyperscanning, the mentioned study is also an ecological study with an audience, and it is worth to include in the introduction.**

 3. The OSF link to the data is broken https://osf.io/yueqk so I cannot guarantee that the data is accessible currently (reference 96 in the manuscript). 

**References, Reference 96. Thank you for noticing this, we contacted OSF and their team helped us to fix the problem. The database is available again.**

 4. The following statement in the discussion is very speculative, and not backed by any references. *"An interesting finding from this study is that brain-to brain synchrony appears to be limited to the higher frequency bands. Although it may seem that music performance requires behavioral coordination across these longer timescales (i.e. musical phrases for example) that may involve delta and theta bands, it is possible that the jazz improvisation performed by the musicians imposed higher demands in higher frequency bands, which are also more localized. Future studies comparing jazz improvisation with other types of music performances that may emphasize longer timescales could elucidate this question."* In  [Farrugia et al. 2021], we did find modulations in the theta and beta oscillations related to subjective states of the improviser, and this was also on "free jazz" (see for example https://www.youtube.com/watch?v=ILhaZYtW8fs&t=390s ). But still, I don't find the statement about linking musical timescales and oscillations frequencies very convincing overall. I'm also not sure what the authors mean by "more localized" in this context. I would recommend removing this paragraph.

**Discussion. The paragraph was removed following this recommendation.**

 5. Authors should refrain from doing direct inferences about neural sources based on scalp electrode positions, such as " *The number of inter-connected channels and ROIs increase when there are more musicians performing. This happened mainly across temporal, occipital and parietal regions, related to auditory, visual and spatial processing*.", or " *posterior (e.g., parietal, temporal and occipital) regions that are involved in the processing of sensory input and are important in interpreting sensory feedback from the external environment*". It's ok to mention interconnected channels and ROIs, but it's impossible to say how much this is related to auditory, visual and spatial processing without doing source localization. Generally speaking, in the results and in the discussion, the authors should stick to channel positions and ROIs at the scalp level, without linking those to brain areas or brain functions.

**In results and discussion sections, we limited to talk about channel position and ROI level. Sentences linking brain areas and functions in results and discussion were removed, for instance in: Discussion, Paragraph 5; Results, Summary of main findings.**

 Minor issues: 

- "Towards the final segments of the performance, there were more ’musical voices’ interacting, " a space is missing before 'musical

**Thank you for noticing, the space was added.**

- A very general question related to one of the limitations that is discussed. The authors do mention that the observed effects might be related to movement artifacts ; is there any statistical analysis that can be presented in this paper to at least show that such a confound is equally "strong" across conditions (e.g. between synchronized and not synchronized performances)
- **Movement artifacts are indeed something to consider in future steps. However, to answer the reviewer comment, Figure 6 notes that when comparing brain synchrony across different conditions, there is a statistical difference at the onset of the events (which were labeled by raters independently).** **If movement artifacts were the only cause for the changes in synchrony, the synchrony changes would appear across all the time window, and not only at the onset.**

# Reviewer report: Reviewer response for version 2

**DOI:** 10.5256/f1000research.147429.r172450

**Published:** 2023-06-02

**Authors:** Stephane Perrey

**Affiliations:** 1 EuroMov Digital Health in Motion, University Montpellier, IMT Mines Ales, Montpellier, France

Thanks to the authors for revising their manuscript satisfactorily. I have no futher major comments to address. This study makes notable step towards conducting neuroscience studies during natural human activities. This case study is methodologically sound and highlights scientific questions about cooperative behaviour and its neural correlates.

Is the case presented with sufficient detail to be useful for teaching or other practitioners?

Yes

Is the work clearly and accurately presented and does it cite the current literature?

Yes

If applicable, is the statistical analysis and its interpretation appropriate?

Yes

Are all the source data underlying the results available to ensure full reproducibility?

Yes

Are the conclusions drawn adequately supported by the results?

Yes

Is the background of the case’s history and progression described in sufficient detail?

Yes

Reviewer Expertise:

Neuroscience and Neuroimaging on the field.

I confirm that I have read this submission and believe that I have an appropriate level of expertise to confirm that it is of an acceptable scientific standard.

# Reviewer report: Reviewer response for version 2

**DOI:** 10.5256/f1000research.147429.r153202

**Published:** 2023-05-18

**Authors:** Guido Orgs

**Affiliations:** 1 Department of Psychology, Goldsmiths, University of London, London, UK

This paper reports a hyper-scanning study on jazz improvisation, using a clever experimental design that dissociates brain synchrony during listening from brain synchrony during joint performance. The authors compute bispectra between musicians during joint improvisation and use musical feature analysis to distinguish brain-to-brain synchrony between synchronised and desynchronised sections of the music. Overall, the study reports greater brain to synchrony in gamma and beta bands during joint performance compared to baseline and greater brain-to brain synchrony during synchronised as compared to desynchronised sections. This is an interesting study that makes important steps towards conducting neuroscience studies in real world environments and during natural human activities such as making music. The study is methodologically strong and raises important questions about cooperative behaviour and its neural correlates, a few more general comments.

1. It would be interesting to see how the bispectral measures compare to more established measures of brain-to-brain synchrony such as PLV or inter-subject correlations. From a methodological perspective it will be important to understand how different measures of neural synchrony relate to specific tasks or activities.
2. How do the authors interpret the lack of brain-to-brain synchrony on lower frequency bands, like theta and delta. Music performance requires behavioural coordination across these longer timescales, i.e. musical phrases for example. It is interesting that brain-to brain synchrony appears to be limited to the higher frequency bands.
3. The authors acknowledge a potential role for movement in mediating synchrony between music performers. Aside from movement, synchronised breathing might be another source of similarity in the performers’ EEG signals, particular in the context of making music together. It would be interesting to replicate the current study with additional psychophysiological and behavioural measures, i.e. EMG or accelerometers to more clearly identify the relative sources of synchrony in the bispectrum.
4. In relation to the previous point, it seems to me that movement and psychophysiological synchrony are not necessarily best understood as artifacts but may well provide an important functional role in supporting joint performance. It would be interesting to see to what extent joint performance and its neural correlates depends on the ability of different modalities, that is seeing and hearing the other players or only hearing or seeing the other players. Such comparisons would provide new insights into how joint performance is actually achieved in musical improvisation.

Overall, this study provides an important step towards better understanding how musicians perform together and which cognitive mechanisms are at play in the process.

Is the case presented with sufficient detail to be useful for teaching or other practitioners?

Yes

Is the work clearly and accurately presented and does it cite the current literature?

Yes

If applicable, is the statistical analysis and its interpretation appropriate?

Yes

Are all the source data underlying the results available to ensure full reproducibility?

Partly

Are the conclusions drawn adequately supported by the results?

Partly

Is the background of the case’s history and progression described in sufficient detail?

Yes

Reviewer Expertise:

Live performance, Mobile EEG, Time-series analyses, Dance, Neuroaesthetics, Joint action and perception.

I confirm that I have read this submission and believe that I have an appropriate level of expertise to confirm that it is of an acceptable scientific standard, however I have significant reservations, as outlined above.

# Response

**Published:** 2023-06-22

**Authors:** Mauricio Ramirez

**Affiliations:** University of Houston, USA

**We thank the reviewer for the time invested in reading and making such useful comments for our work. Below is a response to each of the comments (in bold letters), as well as the locations of the corrections made to the manuscript. All changes are carried out in the next version (Version 3) of the manuscript, which should be published soon.**

1. It would be interesting to see how the bispectral measures compare to more established measures of brain-to-brain synchrony such as PLV or inter-subject correlations. From a methodological perspective it will be important to understand how different measures of neural synchrony relate to specific tasks or activities.

**Introduction, Brain synchrony assessment through hyperscanning, Paragraph 4.** **A paragraph (from the methodological perspective of brain-to-brain synchrony) comparing bispectral measures to the PLV metric was added as suggested.**

1. How do the authors interpret the lack of brain-to-brain synchrony on lower frequency bands, like theta and delta. Music performance requires behavioural coordination across these longer timescales, i.e. musical phrases for example. It is interesting that brain-to brain synchrony appears to be limited to the higher frequency bands.

**Discussion, Paragraph 6. A paragraph was added in the Dicussion section where we discuss our thinking on the appearance of brain synchrony at higher frequency bands, for this particular study case.**

1. The authors acknowledge a potential role for movement in mediating synchrony between music performers. Aside from movement, synchronised breathing might be another source of similarity in the performers’ EEG signals, particular in the context of making music together. It would be interesting to replicate the current study with additional psychophysiological and behavioural measures, i.e. EMG or accelerometers to more clearly identify the relative sources of synchrony in the bispectrum.

**Discussion, Paragraph 17.** **We agree with the reviewer’s comment, and added a statement in the discussion section mentioning that breathing synchrony could be a source of similarity in the EEG signals, and will need to be addressed in future studies.**

1. In relation to the previous point, it seems to me that movement and psychophysiological synchrony are not necessarily best understood as artifacts but may well provide an important functional role in supporting joint performance. It would be interesting to see to what extent joint performance and its neural correlates depends on the ability of different modalities, that is seeing and hearing the other players or only hearing or seeing the other players. Such comparisons would provide new insights into how joint performance is actually achieved in musical improvisation.

**Discussion, Paragraph 18.** **We completely agree with the reviewer’s comment, and added a statement regarding this in the Discussion section.**

# Reviewer report: Reviewer response for version 1

**DOI:** 10.5256/f1000research.135627.r158229

**Published:** 2023-01-20

**Authors:** Stephane Perrey

**Affiliations:** 1 EuroMov Digital Health in Motion, University Montpellier, IMT Mines Ales, Montpellier, France

This study aimed to assess temporal synchronization of EEG signals between three professional musicians interacting during a jazz performance. Some relevant interactions were found through a bispectral analysis. A reflective cooperative intention was observed during the task. This original and pilot study was carried out during a free jazz improvisation tasks of long duration in real-world scenario by expert musicians. This case study is well presented and proposes very interesting thoughts and new perspectives in social neuroscience.

 Authors were able to deal nicely with possible artifacts associated to the movements during such long plays by performing suitable analyses with EEG signals.

 Below you will find some comments for your convenience.

**Introduction:**

- Concerning the following sentence, additional insight offered by the bispectrum, when compared to other neural synchrony metrics, needs to be clarified more. First, what does mean here " *a more complete intuition on phase coupling, resonance, temporal synchronization and non-linear interactions between any analyzed signal pair*". Second, Please highlight the added-value of bispectrum if any.
- The following sentence without reference does not bring more into the rationale of this study and could be removed: " *Musical improvisation can also be considered as an’on the fly’ composition, one that is temporally ubiquitous, spontaneous, and is not restricted by critique.*"
- Jazz performance needs to be defined/characterized in the introduction; different stages do exist.
- " *Brain-to-brain communication*", as an important metric proposed by authors is cited as a shared cognitive state. Please confirm is this is right and how this metric is associated to hyperscanning methods.
- Overall, introduction is delivering many terms around hyperscanning techniques, which makes reading difficult. 
- Please explain/inform on what is a " *creative collaboration*" task, not really defined in the introduction. Thereafter you wrote " *collaborative musical improvisation*". Are you saying the same?
- In the following sentence (end of introduction), since the previous underlined/questioned terms are present, they have to be sufficiently clear. " *The current study examines the neural correlates of brain-to-brain communication of jazz musicians during collaborative musical improvisation through hyperscanning".*
- Mobile EEG can impose movement constraints. This is not so straightforward. Please adjust accordingly.

**Methods**

- Participants. In terms of experiences (years performing jazz together, musical background) for collaborative tasks, P1 and P2 have likely more chance to "interact » better during collaborative musical improvisation; P3 should present some different responses. Does this heterogeneity was voluntary targeted?
- Impedance was set to less than 25 kOhms. It appears relatively high as compared to classic/regular use in the field. Can you provide some methodological reference/guidelines?
- Analysis methods (EEG features, temporal and spatial) are well detailed and are suitable for the data set. Assessment of brain synchrony (maybe state " *brain-to brain communication*") across two ecological sections (synchronized or not) and three participant conditions (active vs passive in a dyad) of jazz performance is relevant and within the scope of Neuroergonomics. Statistical analysis are complete. 

**Results**

- There are many results, some of them being very observational due to the exploratory feature of this case study. However, can authors select the main findings regarding the brain-to brain communication of Jazz musicians during the collaborative musical improvisation sequences?

**Discussion**

- This section presents some first evidence and underlines well some specific intra- inter-brain coupling modulated by musical training and previous experiences. Perspectives and methodological limitations are provided. Body movements as main sources of EEG artefacts should be captured in a further study in order to get synchronisation at the behavioral level associated to their neural correlates. 
- Emotion is likely playing a role in the proposed collaborative musical improvisation task (see doi: 10.3389/fnhum.2022.944241) between the musicians but also with audience, as musicians interact in a jazz performance with a live audience. Please advice. 
- This study included freely-moving expert musicians. **Do you have collected/recorded the body motion of the musicians? Have you any ideas on the kind of movements over the two sequences defined (SP and DP)?Also, it might be surprising to not observe brain synchronization with the motor regions. The ability to synchronize movement with auditory rhythms is relying on motor networks, such as cortical areas, basal ganglia and the cerebellum, which also participate in rhythm perception and movement production (doi: 10.1016/j.neubiorev.2019.12.024). Have you look at these cortical regions?
- Minor: correct " *synchornicity in sensory areas*"

Is the case presented with sufficient detail to be useful for teaching or other practitioners?

Yes

Is the work clearly and accurately presented and does it cite the current literature?

Yes

If applicable, is the statistical analysis and its interpretation appropriate?

Yes

Are all the source data underlying the results available to ensure full reproducibility?

Yes

Are the conclusions drawn adequately supported by the results?

Yes

Is the background of the case’s history and progression described in sufficient detail?

Yes

Reviewer Expertise:

Neuroscience and Neuroimaging on the field.

I confirm that I have read this submission and believe that I have an appropriate level of expertise to confirm that it is of an acceptable scientific standard, however I have significant reservations, as outlined above.

## References

[1] : Positive emotions foster spontaneous synchronisation in a group movement improvisation task. *Front Hum Neurosci* .2022;16: 10.3389/fnhum.2022.944241 944241 10.3389/fnhum.2022.944241 36111209PMC9468250

[2] : Why do we move to the beat? A multi-scale approach, from physical principles to brain dynamics. *Neurosci Biobehav Rev* .2020;112: 10.1016/j.neubiorev.2019.12.024 553-584 10.1016/j.neubiorev.2019.12.024 31846652

# Response

**Published:** 2023-04-24

**Authors:** Mauricio Ramirez

**Affiliations:** University of Houston, USA

**We thank the reviewer for the time invested in reading and making such useful comments for our work. Below is a response to each of the comments (in bold letters), as well as the locations of the corrections made to the manuscript. All changes are carried out in the next version of the manuscript.**

  

- Concerning the following sentence, additional insight offered by the bispectrum, when compared to other neural synchrony metrics, needs to be clarified more. First, what does mean here " *a more complete intuition on phase coupling, resonance, temporal synchronization and non-linear interactions between any analyzed signal pair*". Second, Please highlight the added-value of bispectrum if any.
**Introduction, Page 2, Lines 111-115. The mentioned paragraph was extended to be clearer. References were included as well as some advantages of using the bispectrum metric.** 

- The following sentence without reference does not bring more into the rationale of this study and could be removed: " *Musical improvisation can also be considered as an’on the fly’ composition, one that is temporally ubiquitous, spontaneous, and is not restricted by critique.*"
**Introduction, Page 2, Lines 169-173. The sentence was rephrased, and a new reference from a music composition book was added.**
- Jazz performance needs to be defined/characterized in the introduction; different stages do exist.
**Introduction, Page 3, Lines 176-186. A paragraph adding more detail about jazz and free jazz performance was added with a reference.**
** **

- " *Brain-to-brain communication*", as an important metric proposed by authors is cited as a shared cognitive state. Please confirm is this is right and how this metric is associated to hyperscanning methods.
**Introduction, Page 3, Lines 231-238. A paragraph and a reference were included to add more detail about the use of hyperscanning for assessment of brain-to-brain communication.** 
- Overall, introduction is delivering many terms around hyperscanning techniques, which makes reading difficult. 
**Introduction. Page 1 – 3. Subsections were added to the Introduction to guide the reader throughout the text. No text was removed since in further comments, reviewer suggests that some terms are explained clearly.**
- Please explain/inform on what is a " *creative collaboration*" task, not really defined in the introduction. Thereafter you wrote " *collaborative musical improvisation*". Are you saying the same?
**Introduction, Page 3, Line 230 Yes, they imply the same idea, however the term “collaborative musical improvisation” was used instead, to avoid confusion. **
- In the following sentence (end of introduction), since the previous underlined/questioned terms are present, they have to be sufficiently clear. " *The current study examines the neural correlates of brain-to-brain communication of jazz musicians during collaborative musical improvisation through hyperscanning".*
**Thank you for the comment, we made sure previous terms are clear and present, as suggested.**
- Mobile EEG can impose movement constraints. This is not so straightforward. Please adjust accordingly.
**Introduction, Page 3, Lines 263-264. A clarification on this was added, movement constraints imposed by mobile EEG are less than for other brain activity measuring techniques, for example fMRI scans. **

**Methods**

- Participants. In terms of experiences (years performing jazz together, musical background) for collaborative tasks, P1 and P2 have likely more chance to "interact » better during collaborative musical improvisation; P3 should present some different responses. Does this heterogeneity was voluntary targeted?
**Methods, Page 4, Lines 316-319. It was not targeted on purpose, those were the volunteers that agreed to participate in the study. An observation on this regard was included.**
** **

- Impedance was set to less than 25 kOhms. It appears relatively high as compared to classic/regular use in the field. Can you provide some methodological reference/guidelines?
**Methods, Page 4, Lines 335-339. A reference was added to how this value was selected.**
** **

- Analysis methods (EEG features, temporal and spatial) are well detailed and are suitable for the data set. Assessment of brain synchrony (maybe state " *brain-to brain communication*") across two ecological sections (synchronized or not) and three participant conditions (active vs passive in a dyad) of jazz performance is relevant and within the scope of Neuroergonomics. Statistical analysis are complete. 
**Thank you, we appreciate your comments. **

**Results**

- There are many results, some of them being very observational due to the exploratory feature of this case study. However, can authors select the main findings regarding the brain-to brain communication of Jazz musicians during the collaborative musical improvisation sequences?
**Results, Page 10, Lines 730-744. Main findings were added and highlighted by the end of the results section, in a new subsection. **

**Discussion**

- This section presents some first evidence and underlines well some specific intra- inter-brain coupling modulated by musical training and previous experiences. Perspectives and methodological limitations are provided. Body movements as main sources of EEG artefacts should be captured in a further study in order to get synchronisation at the behavioral level associated to their neural correlates. 
**Discussion, Page 15, Lines 1049-1051.Thank you, this is a great comment, which indeed can be explored in a future study. A note on this regard was added to the discussion section. **
** **

- Emotion is likely playing a role in the proposed collaborative musical improvisation task (see doi: 10.3389/fnhum.2022.944241) between the musicians but also with audience, as musicians interact in a jazz performance with a live audience. Please advice. 
**Discussion, Page 15. Lines 1052-1059. A comment including the emotional context during musical improvisation was included as suggested.**
** **

- This study included freely-moving expert musicians. **Do you have collected/recorded the body motion of the musicians? Have you any ideas on the kind of movements over the two sequences defined (SP and DP)?Also, it might be surprising to not observe brain synchronization with the motor regions. The ability to synchronize movement with auditory rhythms is relying on motor networks, such as cortical areas, basal ganglia and the cerebellum, which also participate in rhythm perception and movement production (doi: 10.1016/j.neubiorev.2019.12.024). Have you look at these cortical regions?
**Only head motion was collected, but not analyzed in this study. Indeed will be included as future steps. The auditory-motor synchronization in the references talk about such functional network within one person. However, when analyzing our data, synchronization between motor areas and motor-auditory were not observed. We believe this was due to the individuality of movement in the production of sounds (motor areas not in synch), however the perceptual information and processes were shared among musicians, therefore the synchrony in temporal, parietal, and occipital regions (perceptual areas in synch).**
** **

- Minor: correct " *synchornicity in sensory areas*"
**This error was fixed. Discussion, Page 12, Lines 838.**
